# Genes for asparagine metabolism in *Lotus japonicus*: differential expression and interconnection with photorespiration

**DOI:** 10.1186/s12864-017-4200-x

**Published:** 2017-10-12

**Authors:** Margarita García-Calderón, Carmen M. Pérez-Delgado, Alfredo Credali, José M. Vega, Marco Betti, Antonio J. Márquez

**Affiliations:** Departamento de Bioquímica Vegetal y Biología Molecular, Facultad de Química, C/ Profesor García González, 1, 41012 Sevilla, Spain

**Keywords:** Asparaginase genes, Asparagine synthetase genes, *Lotus japonicus*, Serine-glyoxylate aminotransferase genes

## Abstract

**Background:**

Asparagine is a very important nitrogen transport and storage compound in plants due to its high nitrogen/carbon ratio and stability. Asparagine intracellular concentration depends on a balance between asparagine biosynthesis and degradation. The main enzymes involved in asparagine metabolism are asparagine synthetase (ASN), asparaginase (NSE) and serine-glyoxylate aminotransferase (SGAT). The study of the genes encoding for these enzymes in the model legume *Lotus japonicus* is of particular interest since it has been proposed that asparagine is the principal molecule used to transport reduced nitrogen within the plant in most temperate legumes.

**Results:**

A differential expression of genes encoding for several enzymes involved in asparagine metabolism was detected in *L. japonicus*. ASN is encoded by three genes, *LjASN1* was the most highly expressed in mature leaves while *LjASN2* expression was negligible and *LjASN3* showed a low expression in this organ, suggesting that *LjASN1* is the main gene responsible for asparagine synthesis in mature leaves. In young leaves, *LjASN3* was the only ASN gene expressed although at low levels, while all the three genes encoding for NSE were highly expressed, especially *LjNSE1*. In nodules, *LjASN2* and *LjNSE2* were the most highly expressed genes, suggesting an important role for these genes in this organ. Several lines of evidence support the connection between asparagine metabolic genes and photorespiration in *L. japonicus*: a) a mutant plant deficient in *LjNSE1* showed a dramatic decrease in the expression of the two genes encoding for SGAT; b) expression of the genes involved in asparagine metabolism is altered in a photorespiratory mutant lacking plastidic glutamine synthetase; c) a clustering analysis indicated a similar pattern of expression among several genes involved in photorespiratory and asparagine metabolism, indicating a clear link between *LjASN1* and *LjSGAT* genes and photorespiration.

**Conclusions:**

The results obtained in this paper indicate the existence of a differential expression of asparagine metabolic genes in *L. japonicus* and point out the crucial relevance of particular genes in different organs. Moreover, the data presented establish clear links between asparagine and photorespiratory metabolic genes in this plant.

**Electronic supplementary material:**

The online version of this article (10.1186/s12864-017-4200-x) contains supplementary material, which is available to authorized users.

## Background

Asparagine is a very important nitrogen transport and storage compound in plants due to its high nitrogen/carbon ratio and stability. In fact, asparagine is the major nitrogen transport compound in the xylem from the root to the leaves and in the phloem from the leaves to the developing seed in a range of plants, particularly in temperate legumes [1]. This is the case of the model legume *Lotus japonicus*, where it was shown that asparagine can account for 86% of the nitrogen flux from root to shoot when nitrogen is not limiting [2].

Asparagine intracellular concentration depends on a balance between asparagine biosynthesis and degradation. The main route for asparagine biosynthesis in plants is mediated by the enzyme asparagine synthetase (ASN, EC 6.3.5.4), which catalyzes the ATP-dependent transfer of the amide group of glutamine to aspartate yielding asparagine in the presence of magnesium. Most plant species contain two or more ASN genes mainly grouped into two phylogenetic clades, named class-I and class-II [3, 4], whose expression is subjected to metabolic control. Class-I genes are usually negatively regulated by light or sugars whereas class-II genes are not repressed by light [5, 6]. Besides light and sugars, nitrogen source and availability are other factors that regulate ASN expression [6–8]. This fine regulation allows the plants to have asparagine available at specific phases of development, such as nitrogen mobilization in germinating seeds, ammonium (re)assimilation in leaves and nitrogen remobilization from senescent leaves to developing seeds [3, 9, 10]. The role of a specific asparagine synthetase isoform in primary ammonium assimilation has been also recently investigated [11].

On the other hand, two pathways for asparagine catabolism have been established in plants. The main enzyme for asparagine degradation is asparaginase (NSE, EC 3.5.1.1) that catalyzes the hydrolysis of asparagine to yield aspartate and ammonia, which is subsequently reassimilated by glutamine synthetase [12–15]. The other asparagine degradation route involves the transamination of asparagine in the presence of glyoxylate to yield 2-oxosuccinamate and glycine, catalyzed by asparagine-glyoxylate aminotransferase [16].

Two subfamilies of asparaginases have been described, corresponding to biochemical subtypes defined on the basis of their dependence for K^+^: K^+^-dependent and K^+^-independent asparaginases [13, 14, 17, 18]. The exact meaning of the potassium dependence in asparaginases constitutes an interesting topic of research [14, 19]. Despite of their separate classification, the two groups of plant asparaginases share significant levels (about 60%) of sequence identity.

Asparagine aminotransferase appears to be the same protein as the peroxisomal photorespiratory enzyme serine-glyoxylate aminotransferase (SGAT, EC 2.6.1.45), based on its substrate preference and subcellular localization [16, 20–24]. The 2-oxosuccinamate produced by this enzyme is then reduced to hydroxysuccinamate and subsequently deamidated to yield malate or, alternatively, to oxalacetate and ammonia, by the enzyme omega-amidase [25]. In plants, asparagine transamination catalyzed by SGAT has been proposed to be involved in photorespiration as an input of external nitrogen into the photorespiratory cycle [25–27]. However, SGAT enzymes have been also shown to use serine or alanine efficiently as substrate, besides asparagine [16].

In the present paper the pattern of expression of asparagine metabolism genes was examined in different organs and under different conditions (e.g. light and dark) in *L. japonicus* plants. In addition, the connection between asparagine metabolism genes and photorespiration was also analyzed using two different mutants from this plant. On the one hand, a mutant deficient in asparaginase, called *Ljnse1–4,* which accumulates high levels of asparagine [15]*.* On the other hand, a photorespiratory mutant called *Ljgln2–2*, deficient in the plastidic isoform of glutamine synthetase (GS2), which is the enzyme that reassimilates the NH_4_^+^ produced by the photorespiratory cycle [28, 29]. The expression levels of different genes involved in asparagine metabolism and in the photorespiratory pathway were determined by quantitative RT-PCR in leaves using WT (wild type) and both mutant genotypes. A global transcriptomic analysis was also carried out to further investigate the interconnection between asparagine metabolism and photorespiratory metabolism genes in this plant. The data presented in this paper indicate the existence of a differential expression of asparagine metabolism genes in *L. japonicus* and point out the crucial relevance of particular genes in different organs and/or processes related with asparagine metabolism and the photorespiratory pathway in this plant.

## Methods

### Plant materials and treatments

*L. japonicus* (Regel) Larsen cv. Gifu was initially obtained from Prof. Jens Stougaard (University of Aarhus, Denmark) and then self-propagated at the University of Seville. Plant seeds were scarified and surface-sterilized, germinated in Agar/Horned Petri dishes, and transferred to pots using vermiculite as solid support. Five seedlings were placed in each pot and grown during 35 days in a chamber under 16/8 h day/night, 20/18 °C, with a photosynthetic photon flux density of 250 μmol m^−2^·s^−1^ and a constant humidity of 70%. Plants were watered with Hornum nutrient solution [30]. Plants grown in symbiosis with nitrogen-fixing bacteria were inoculated with *Mesorhizobium loti*, as described previously by García-Calderón et al. [31].

The *Ljgln2–2* mutant used in this work was previously isolated from a photorespiratory mutant screening [28, 32, 33]. The mutant offspring of two consecutive backcrosses of *Ljgln2–2* into the WT background was employed. In addition, for other experiments, a mutant in the *LjNSE1* gene of *L. japonicus* was used, called *Ljnse1–4*, that was previously isolated by TILLING (Targeted Induced Local Lesions IN Genomes) as described by Credali et al. [15].

### RNA extraction and qRT-PCR

Tissues from *L. japonicus* plants were harvested and immediately frozen in liquid nitrogen, homogenized with a mortar and pestle, and kept at −80 °C until use. Total RNA was isolated using the hot borate method [34]. The integrity and concentration of the RNA preparations were checked using an Experion bioanalyzer (Bio-Rad) with RNA StdSens chips and a NanoDrop ND-1000 (NanoDrop Technologies), respectively. RNA extraction was carried out using three independent biological samples for each genotype/condition/tissue. A biological replicate consisted of tissue pooled from five plants grown in the same pot.

For qRT-PCR analysis, total RNA was treated with the TURBO DNA-free Kit (Ambion). Reverse transcription was carried out using SuperScript III reverse transcriptase (Invitrogen), oligo-dT and RNAsin RNAse inhibitor (Promega). DNA contamination and RNA integrity were checked by performing the qRT-PCR reactions with oligonucleotides that amplified an intron in the *L. japonicus* hypernodulation aberrant root formation (*LjHAR1*) gene and the 5′ and 3′ ends of the *L. japonicus* glyceraldehyde-3-phosphate dehydrogenase respectively (using the oligonucleotide pairs *LjGAPDH5’* and *LjGAPDH3’*). qRT-PCR analysis was performed as described by Pérez-Delgado et al. [35] using the SensiFAST™ SYBR® No-ROX Kit (Bioline) and a LightCycler® 480 II thermal cycler (Roche).

Ct values were determined using the LightCycler 480 software version 1.5.0 and the 2^-Ct^ values were standardized by dividing them by geometric mean of the 2^-Ct^ values of four different housekeeping genes: *L. japonicus* glycosylphosphatidyl inositol (*LjGPI*)-anchored protein (Lj3g3v1933150.1), *L. japonicus* protein phosphatase 2A (*LjPp2A*) (Lj2g3v0742070.1), *L. japonicus* ubiquitin carrier protein 10 (*LjUbc10*) (Lj1g3v2063210.1) and *L. japonicus* polyubiquitin 4 (*LjUbq*) (Lj5g3v2060710.1) that were selected among the most stably expressed genes in plants [36], and that were found to be suitable for gene expression analysis also in *L. japonicus* [34, 35, 37]. A list of all oligonucleotides used is provided in Additional file 1: Table S1.

### Clustering analysis of qRT-PCR data

For hierarchical clustering of qRT-PCR data the transcript levels of WT plants under high CO_2_ conditions were taken as 1 and the differences between the log_2_ of relative expression levels of *Ljgln2–2* and WT are presented. Hierarchical clustering of the data was performed using the Multiexperiment Viewer software version 4.9.0 [38] with optimized gene leaf order and complete linkage clustering algorithm. The expression data of some genes of nitrogen and photorespiratory metabolism were taken from Pérez-Delgado et al. [39].

### Clustering analysis of microarray data

- *Microarray data collection and preprocessing*

The microarray data used in this work were obtained from the experiments published by: Sánchez et al. [34], Pérez-Delgado et al. [39], Høgslund et al. [40], Díaz et al. [41], Sánchez et al. [42], and Betti et al. [43]. These experiments have a total of 84 different conditions (samples) and 240 hybridizations. CEL files of these experiments are available in the public microarrays database ArrayExpress [44]. The code numbers of the experiments are: E-MEXP-1204, E-TABM-715, E-MEXP-2344, E-MEXP-2690, E-MEXP-1726, E-MEXP-3710 and E-MEXP-3603. Background correction and normalization of the raw data sets were performed using Robust MultiChip Analysis (RMA) implemented in “affy” R package [45].

b). *Clustering analysis*

For the clustering analysis the log_2_ of the relative gene expression levels in all the different transcriptional experiments considered was used. Hierarchical clustering of transcriptomic data was carried out with Expander software version 7.1 [46, 47] using the complete linkage option.

## Results

### Genes for asparagine metabolism in *L. japonicus*

Three genes encoding for asparagine synthetase were found in the *L. japonicus* genome by searching the available databases [48]: *LjASN1* (accession number, Lj2g3v2291670.1), *LjASN2* (accession number, Lj0g3v0295349.1) and *LjASN3* (accession number, Lj0g3v0361789.1). According to phylogenetic analysis *LjASN1* and *LjASN2* belong to class I asparagine synthetases while *LjASN3* belong to class II (Additional file 2: Figure S1). The genomic sequences of all these genes were analyzed. Thirteen, thirteen and fourteen exons were found in the genes encoding for LjASN1, LjASN2 and LjASN3 respectively (Additional file 3: Figure S2). The size of exons is similar comparing *LjASN1* and *LjASN2*, but different for some particular exons in *LjASN3*.

In the case of asparaginase, three genes were identified: *LjNSE1* (accession number, Lj5g3v0296030.1), *LjNSE2* (accession number, Lj4g3v1736160.1), and *LjNSE3* (accession number, Lj0g3v0303539.1) in the *L. japonicus* genome. Although the size of the genes was different, *LjNSE1* and *LjNSE2* genes had a similar structure composed of four exons, while five exons were found in the *LjNSE3* gene (Additional file 3: Figure S2). According to the deduced amino acid sequence data *LjNSE1* and *LjNSE3* encode for K^+^- dependent asparaginases while *LjNSE2* encodes for a K^+^- independent asparaginase, based on the previous analysis carried out for LjNSE1 and LjNSE2 enzymes [14].

Two genes encoding for serine-glyoxylate aminotransferase, *LjSGAT1* (accession number, Lj6g3v0937010.1) and *LjSGAT2* (accession number, Lj2g3v3058530.1), were also found in the *L. japonicus* genome. The gene structure and the number of exons were also similar between these two *SGAT* genes although both genes showed a different size.

### Transcriptional analysis of LjASN genes

**Fig. 1:**
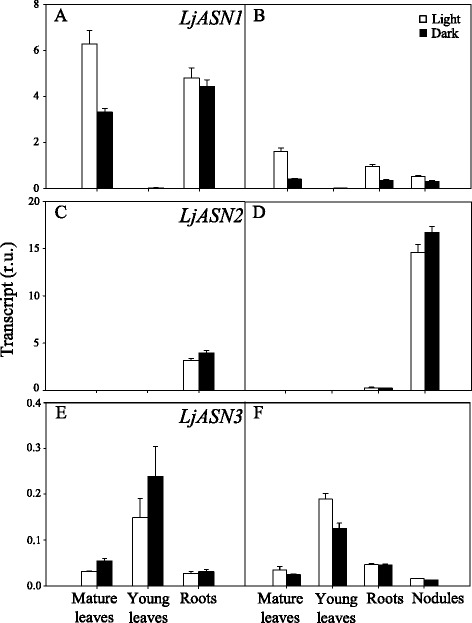
qRT-PCR analysis of LjASN genes expression in different tissues of *L. japonicus* *.* Plants were grown for 6 weeks and irrigated with Hornum medium containing NH_4_NO_3_ (**a**, **c**, **e**), or inoculated with *M. loti* and irrigated with Hornum medium without nitrogen (**b**, **d**, **f**). The samples were harvested at 4 h after the beginning of the light or dark period for the quantification of transcripts. Transcript levels are reported as relative units (r.u.). Data are the mean ± SE of three independent biological replicates

The levels of expression of the three genes encoding for ASN were determined by measuring by quantitative RT-PCR the mRNA levels present in different organs of *L. japonicus* plants supplied with external nitrogen (NH_4_NO_3_) or under purely symbiotic conditions. For comparative purposes, the measurements were carried out from samples taken either under light or dark conditions (Fig. 1). Under both N nutritions *LjASN1* was highly expressed in mature leaves and roots, but its expression was insignificant in young leaves. The expression of *LjASN1* was clearly up-regulated by light (about 2-fold) in mature leaves (Fig. 1A, B). In contrast, *LjASN2* gene expression was negligible in leaves (Fig. 1C, D), while *LjASN3* showed very low levels of expression compared to *LjASN1* in this tissue (Fig. 1E, F). These results indicate that the expression of the *LjASN1* gene must be crucially important for the biosynthesis of asparagine in mature leaves of *L. japonicus*. In young leaves of this plant, expression was only detectable for the *LjASN3* gene, although its expression levels were quite low compared to the other *LjASN* genes in the other tissues examined.

All the three genes encoding for ASN were expressed in roots, particularly *LjASN1* and *LjASN2*, although they were not generally affected by light (Fig. 1). This situation contrasts with the expression levels detected in nodules, where *LjASN2* was the most highly expressed gene. Interestingly, very little expression of this particular *LjASN2* gene was detected in the other organs examined from nodulated plants compared to nodules (Fig. 1D). These results indicate an important role of the *LjASN2* gene for asparagine biosynthesis in nodules.

### Transcriptional analysis of LjNSE and LjSGAT genes

**Fig. 2:**
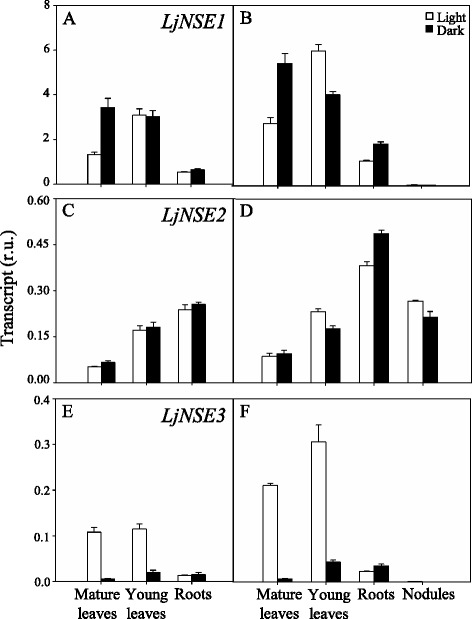
qRT-PCR analysis of LjNSE genes expression in different tissues of *L. japonicus*. Plants were grown for 6 weeks and irrigated with Hornum medium containing NH_4_NO_3_ (**a**, **c**, **e**), or inoculated with *M. loti* and irrigated with Hornum medium without nitrogen (**b**, **d**, **f**). The samples were harvested at 4 h after the beginning of the light or dark period for the quantification of transcripts. Transcript levels are reported as relative units (r.u.). Data are the mean ± SE of three independent biological replicates

The expression levels of *LjNSE* genes were also comparatively determined in nodulated plants and plants supplied with external N. *LjNSE1* was the most highly expressed in all the plant tissues analysed with the exception of nodules (Fig. 2). Interestingly, the expression of this gene was stimulated by dark (2-fold) in mature leaves (Fig. 2A, B). The fact that this gene has more than 10-fold higher expression than the other *LjNSE* genes in leaves, suggests that *LjNSE1* is the main gene involved in asparagine catabolism in this organ. In contrast, the *LjNSE2* transcript was detected in all plant tissues analyzed (Fig. 2C, D) and it was practically the only *NSE* gene expressed in nodules, thus suggesting a crucial role for this gene in asparagine degradation in this organ (Fig. 2D). It is worth noting that, the expression of *LjNSE3* was significantly lower than that of *LjNSE1* in leaves, roots and nodules (Fig. 2E, F), but a significant light-dependent expression was observed both in mature and young leaves (Fig. 2E, F).

**Fig. 3:**
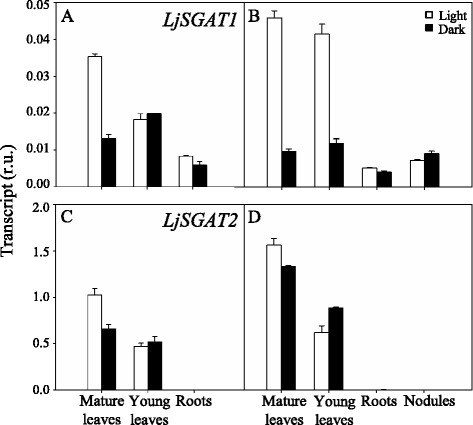
qRT-PCR analysis of LjSGAT genes expression in different tissues of *L. japonicus*. Plants were grown for 6 weeks and irrigated with Hornum medium (**a**, **c**), containing NH_4_NO_3_ or inoculated with *M. loti* and irrigated with Hornum medium without nitrogen (**b**, **d**). The samples were harvested at 4 h after the beginning of the light or dark period for the quantification of transcripts. Transcript levels are reported as relative units (r.u.). Data are the mean ± SE of three independent biological replicates

With respect to the expression of the genes encoding for SGAT, *LjSGAT2* was the most highly expressed gene in young and mature leaves. Its level of expression was 25–30 fold higher in comparison with *LjSGAT1* (Fig. 3). However, the *LjSGAT2* transcript was undetectable in roots and nodules (Fig. 3C, D). Interestingly, although the *LjSGAT1* gene showed a much lower expression, this gene was found to be expressed in all the organs analysed: leaves, roots and nodules, and it was highly light-induced in leaves (Fig. 3A, B).

### A mutant deficient in NSE1 asparaginase shows a dramatic diminution of the transcript levels of LjSGAT genes

**Fig. 4:**
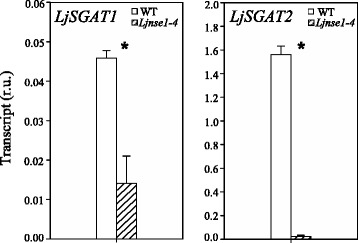
qRT-PCR analysis of LjSGAT genes expression in leaves of WT and Ljnse1–4 mutant plants. *L. japonicus* plants were grown for 6 weeks under normal air conditions. Leaves were harvested at 4 h after the beginning of the light period. Transcript levels are reported as relative units (r.u.). Data are the mean ± SE of three independent biological replicates. * indicates significant differences between WT and *Ljnse1–4* mutant plants as determined by Student’s test (*P* < 0.05)

The possible changes in expression of asparagine metabolism genes were also examined in a TILLING mutant available, called *Ljnse1–4,* which was specifically affected in LjNSE1 asparaginase and accumulated high levels of asparagine [15]. In the present paper no changes were detected in the expression levels of asparagine synthetase and asparaginase genes between WT and *Ljnse1–4* mutant plants under all the conditions considered. However, a dramatic reduction of the transcript levels of *LjSGAT1* and *LjSGAT2* was detected in leaves of the *Ljnse1–4* mutant compared to the WT plants (Fig. 4) in spite of the fact that no changes in expression levels of *LjSGAT* genes were observed in roots and nodules (not shown). It can be concluded that the deficiency of LjNSE1 has a very important influence in the expression of the genes encoding for SGAT. Considering that SGAT has been previously associated to the N photorespiratory cycle in other plant species [16, and references therein], the results obtained could be taken as an indication of the existence of a close relationship between the expression of genes for asparagine metabolism and photorespiratory genes in *L. japonicus*.

### Changes in the transcription of genes for asparagine metabolism in a Ljgln2 photorespiratory mutant

The previous results gave rise to the study of the possible connection between asparagine metabolism and the photorespiratory process in *L. japonicus*. For this purpose, the plants were grown under a CO_2_-enriched atmosphere (0.7% *v*/v) where photorespiration is suppressed and transferred to a normal air atmosphere (about 0.04% CO_2_) where photorespiration is active. A *L. japonicus* photorespiratory mutant, *Ljgln2–2*, deficient in the plastidic isoform of glutamine synthetase (GS_2_) that was previously isolated and characterized at the molecular level in our laboratory [28, 32] was also included in our study to determine the influence of the impairment of the photorespiratory cycle on asparagine metabolism. It has been previously shown that this plant accumulated significant amounts of ammonium and asparagine when transferred from high CO_2_ to air as a result of GS_2_ deficiency [39].

**Fig. 5:**
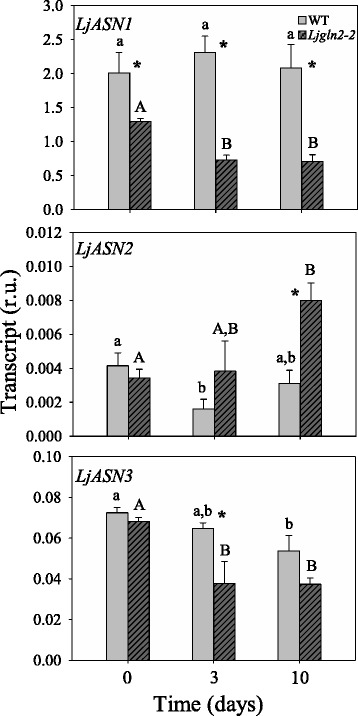
qRT-PCR analysis of LjASN genes expression in leaves of WT and Ljgln2–2 mutant plants***.*** *L. japonicus* plants grown for 35 days in high CO_2_ (time zero) were transferred to normal CO_2_ conditions for the indicated periods of time. Leaves were harvested at the indicated time points. Transcript levels are reported as relative units (r.u.). Data are the mean ± SE of three independent biological replicates. * indicates significant differences between WT and *Ljgln2–2* mutant plants; a, b indicate significant differences between CO_2_ and air conditions at the indicated time points for WT plants; A, B indicate significant differences between CO_2_ and air conditions at the indicated time points for *Ljgln2–2* plants as determined by Student’s test (*P* < 0.05)

The expression levels of genes involved in asparagine metabolism in the WT and mutant plants were determined at different time points after the transfer from high CO_2_ (suppressed photorespiration) atmosphere to normal air (active photorespiration) conditions. Considering that photorespiration takes place exclusively in leaves, the analyses were only carried out in this organ. The expression levels of *LjASN1* were similar in high CO_2_ and air conditions in WT plants (Fig. 5). However, *LjASN1* expression levels diminished significantly in the *Ljgln2–2* mutant after the transfer to normal air (Fig. 5). On the other hand, *LjASN2* showed a slight diminution in WT plants after the transfer to normal air, recovering after 10 days under photorespiratory conditions. By contrast, the levels of expression of the *LjASN2* gene showed a remarkable increase in *Ljgln2–2* mutant plants after the transfer to active photorespiratory conditions (Fig. 5). *LjASN3* gene expression diminished both in WT and *Ljgln2–2* plants after the transfer to air, and this diminution was more marked in the mutant genotype.

**Fig. 6:**
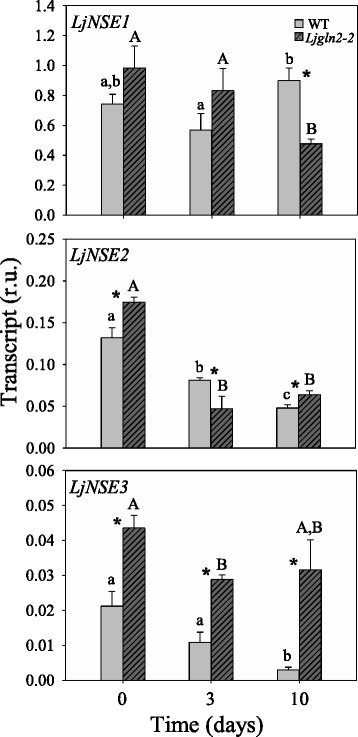
qRT-PCR analysis of LjNSE genes expression in leaves of WT and Ljgln2–2 mutant plants. *L. japonicus* plants grown for 35 days in high CO_2_ (time zero) were transferred to normal CO_2_ conditions for the indicated periods of time. Leaves were harvested at the indicated time points. Transcript levels are reported as relative units (r.u.). Data are the mean ± SE of three independent biological replicates. * indicates significant differences between WT and *Ljgln2–2* mutant plants; *a*, *b*, *c*: indicate significant differences between CO_2_ and air conditions at the indicated time points for WT plants; A, B: indicate significant differences between CO_2_ and air conditions at the indicated time points for *Ljgln2–2* plants as determined by Student’s test (*P* < 0.05)

Several differences were also detected between WT and *Ljgln2–2* photorespiratory mutant plants in regards to the expression of asparaginase genes. *LjNSE1* expression decreased significantly after 10 days of the transfer to air in the mutant but not in the WT (Fig. 6). *LjNSE2* expression levels decreased strongly both in WT and *Ljgln2–2* mutant plants. *LjNSE3* expression levels gradually decreased in the WT after the transfer to normal air and were also significantly diminished in the mutant, but only at day 3 (Fig. 6).

**Fig. 7:**
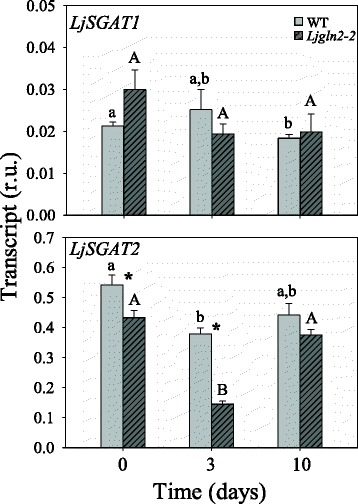
qRT-PCR analysis of LjSGAT genes expression in leaves of WT and Ljgln2–2 mutant plants. *L. japonicus* plants grown for 35 days in high CO_2_ (time zero) were transferred to normal CO_2_ conditions for the indicated periods of time. Leaves were harvested at the indicated time points. Transcript levels are reported as relative units (r.u.). Data are the mean ± SE of three independent biological replicates. * indicates significant differences between WT and *Ljgln2–2* mutant plants; a, b: indicate significant differences between CO_2_ and air conditions at the indicated time points for WT plants; A, B: indicate significant differences between CO_2_ and air conditions at the indicated time points for *Ljgln2–2* plants as determined by Student’s test (*P* < 0.05)

The expression levels of *LjSGAT* genes were also determined in both genotypes. No major changes were observed in the expression levels of *LjSGAT1* either in WT or mutant plants. However, the level of expression of the *LjSGAT2* gene was 15-fold higher than the expression of *LjSGAT1* and decreased substantially after 3 days of the transfer of plants from high CO_2_ to air conditions, particularly in the mutant plants, followed by some recovery after 10 days under air atmosphere (Fig. 7).

**Fig. 8:**
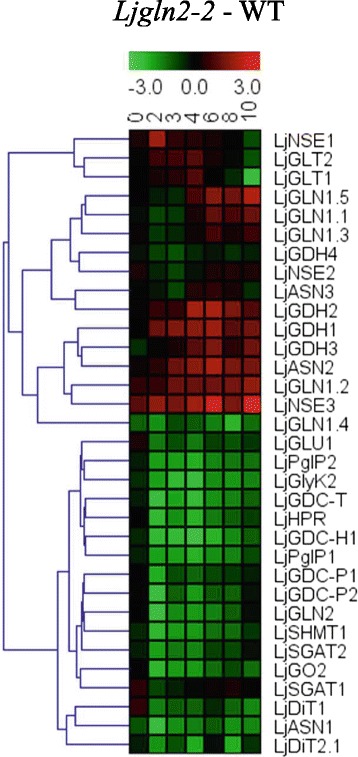
Hierarchical clustering analysis of quantitative RT-PCR data for photorespiratory and nitrogen metabolism genes**.** Transcript levels were determined at the indicated time periods after the transfer of WT and *Ljgln2–2* mutant plants from CO_2_-enriched (time zero) to normal CO_2_ conditions. Relative transcript levels of WT plants under CO_2_-enriched atmosphere were taken as 1. Data are presented as the log2 of the difference of transcript levels between WT and *Ljgln2–2*. The genes considered and their corresponding accession numbers according to the Kazusa database are: asparagine synthetase (*LjASN1*, Lj2g3v2291670.1; *LjASN2*, Lj0g3v0295349.1; *LjASN3*, Lj0g3v0361789.1); asparaginase (*LjNSE1*, Lj5g3v0296030.1; *LjNSE2*, Lj4g3v1736160.1; *LjNSE3*, Lj0g3v0303539.1); serine hydroxymethyltransferase (*LjSHMT1*, Lj2g3v1467880.1); ferredoxin-dependent GOGAT (*LjGLU1*, Lj1g3v4154900.1); NADH-GOGAT (*LjGLT1*, LjSGA_035611.1*; *LjGLT2*, LjSGA_037992.1*); serine:glyoxylate aminotransferase (*LjSGAT1*, Lj6g3v0937010.1; *LjSGAT2*, Lj2g3v3058530.1); plastidic glutamine synthetase (*LjGLN2*, Lj6g3v1887800.1); cytosolic glutamine synthetase (*LjGLN1.1*, Lj2g3v0658180.1; *LjGLN1.2*, Lj6g3v0410490.1; *LjGLN1.3*, Lj0g3v0335159.1; *LjGLN1.4*, LjSGA_058827.1*; *LjGLN1.5*, LjSGA_019428.1*); glutamate dehydrogenase (*LjGDH1*, Lj1g3v3975110.1; *LjGDH2*, Lj4g3v1212370.1; *LjGDH3*, Lj2g3v1988990.1; *LjGDH4*, Lj0g3v0102829.1); hydroxypyruvate reductase (*LjHPR*, Lj5g3v2242500.1); glycine decarboxylase (*LjGDC-H1*, Lj4g3v0654460.1; *LjGDC-P1*, chr5.CM0019.20.r2.m*; *LjGDC-P2*, chr5.LjT34K16.170.r2.m*; *LjGDC-T*, Lj6g3v1849480.1); glycerate kinase (*LjGlyK2*, Lj3g3v2247080.1); glycolate oxidase (*LjGO2*, Lj3g3v1048900.2); plastidic dicarboxylate transporter (*LjDiT1*, chr5.CM0089.610.r2.d*; *LjDiT2.1*, Lj6g3v2204740.1) and phosphoglycolate phosphatase (*LjPglP1*, Lj1g3v2842370.1; *LjPglP2*, Lj6g3v1708420.1). The gene accession numbers are reported according to the version 3.0 of the *L. japonicus* genome in the Kazusa database, except in the cases indicated with an asterisk where the version 2.5 of the genome was used

Noteworthy, the trend of transcriptional regulation observed for *LjASN1* and *LjSGAT2* in the *Ljgln2–2* mutant was found to be quite similar to the one reported in a previous work for most of the photorespiratory genes [39]. A sudden drop in transcript levels was observed upon the transfer from high CO_2_ (suppressed photorespiration) to normal air (active photorespiration) atmosphere, followed by a recovery in some cases of the transcript levels. For this reason, a hierarchical clustering was carried out based on the transcript levels determined at different times of the transfer to air for the different asparagine metabolism genes that were analyzed in the present work, together with those of all known photorespiratory genes and some other nitrogen metabolism genes from *L. japonicus*. Two main clusters of genes were obtained: one that contained most genes for N metabolism (upper cluster in Fig. 8) and another one (lower one in Fig. 8) that contained most photorespiratory genes. Very interestingly, *LjASN1, LjSGAT1* and *LjSGAT2* genes clustered together with the photorespiratory genes. On the other hand, *LjASN2*, *LjASN3, LjNSE1*, *LjNSE2* and *LjNSE3* clustered together with the genes encoding for other enzymes of nitrogen metabolism (Fig. 8). Therefore we conclude that among all asparagine metabolism genes analyzed in the present paper, *LjASN1* and both *LjSGAT1* and *LjSGAT2* genes are those most likely connected with photorespiratory metabolism in *L. japonicus* plants.

### Transcriptomic analysis of asparagine metabolism genes

**Fig. 9:**
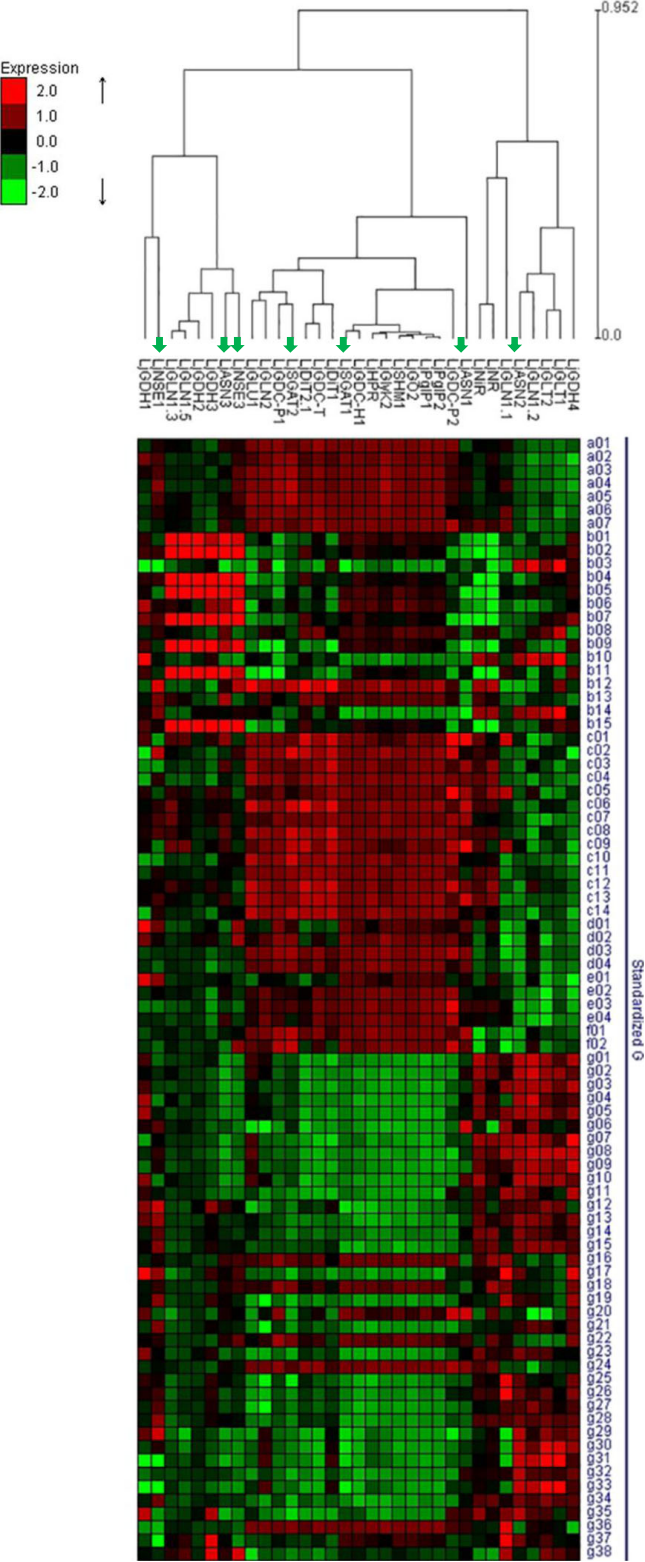
Clustering analysis of transcript levels of asparagine metabolism genes and photorespiratory and N metabolism genes. The clustering analysis was carried out with the Expander software using complete linkage. The mean of the expression level of each gene in all the samples analyzed was calculated and transformed in a log_2_ before clustering analysis. In the color panels, each *vertical line* represents a single gene, and the color of the line indicates the expression level (in a log scale) of the gene relative to a specific sample: high expression in *red*, low expression in *green.* The photorespiratory genes and genes of nitrogen metabolism present in the clustering image are: asparagine synthetase (*LjASN1*, *LjASN2* and *LjASN3*); asparaginase (*LjNSE1* and *LjNSE3*); nitrate reductase (*LjNR*, Lj0g3v0006719.1); nitrite reductase (*LjNiR*, Lj4g3v0588830.1); serine hydroxymethyltransferase (*LjSHM1*)*;* ferredoxin-dependent glutamate synthase (*LjGLU1*); NADH-dependent glutamate synthase (*LjGLT1* and *LjGLT2*); serine:glyoxylate aminotransferase (*LjSGAT1* and *LjSGAT2*); plastidic glutamine synthetase (*LjGLN2)*; cytosolic glutamine synthetase (*LjGLN1.1*, *LjGLN1.2, LjGLN1.3* and *LjGLN1.5*) glutamate dehydrogenase (*LjGDH1*, *LjGDH2*, *LjGDH3* and *LjGDH4*); hydroxypyruvate reductase (*LjHPR*); glycine decarboxylase (*LjGDC-H1*, *LjGDC-P1*, *LjGDC-P2* and *LjGDC-T*); glycerate kinase (*LjGlyK2*); glycolate oxidase (*LjGO2*); plastidic dicarboxylate transporter (*LjDiT1* and *LjDiT2.1*) and phosphoglycolate phosphatase (*LjPglP1* and *LjPglP2)*. The accession numbers of the genes mentioned previously (Fig. 8) can be found in the corresponding figure legend

The pattern of expression of the different genes involved in asparagine metabolism were comparatively examined together with other genes for photorespiratory metabolism and nitrogen metabolism, using for this purpose an integration of all the transcriptomic data available for *L. japonicus* from 84 different physiological conditions and/or tissues (Additional file 4: Table S2). Figure 9 shows a clustering analysis carried out from all these transcriptomic data. A close link was obtained between *LjASN1* gene expression and the expression of most of the photorespiratory metabolism related genes. This was also the case for *LjSGAT1* and *LjSGAT2* genes, which were associated too to *LjASN1* and other photorespiratory genes. Therefore, the transcriptomic analysis carried out confirms again the results previously shown in this paper that establish a clear link between *LjASN1* and *LjSGAT1* and *LjSGAT2* genes with photorespiratory metabolism in *L. japonicus*. In contrast, the pattern of expression of the *LjASN2* gene was more closely associated with a completely different set of genes, mainly encoding for proteins involved in nitrogen assimilation such as nitrate reductase (NR), nitrite reductase (NiR), cytosolic glutamine synthetase (GS1) or NADH-dependent glutamate synthase (NADH-GOGAT). Moreover, it was also shown that the *LjASN2* gene was more highly expressed in roots and nodules than in leaves, as deduced from the corresponding transcriptomic data available from these organs. Consequently, the results obtained from the analysis of the expression of the *LjASN2* gene from an ample set of transcriptomic data are in agreement with the previous results shown in the present paper.

## Discussion

**Fig. 10:**
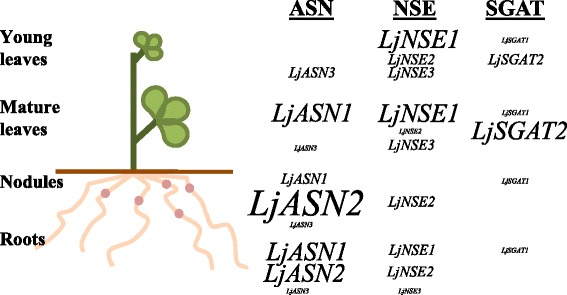
Differential expression of the different genes encoding for LjASN, LjNSE and LjSGAT in *L. japonicus* **.** The size of the lettering reflects the relative abundance of the different genes analyzed in the paper. Genes mentioned in this figure are the key genes for the following biochemical pathways: LjASN, asparagine biosynthesis; LjNSE, asparagine degradation; LjSGAT, photorespiration/transamination

The results obtained in the present paper indicate the existence of a differential expression of various genes involved either in asparagine biosynthesis or degradation in this plant. Figure 10 summarizes in a schematic form the main results obtained in regard to the different pattern of expression found for asparagine synthetase, asparaginase, and *SGAT* genes in different tissues such as young or mature leaves, nodules and roots. The results obtained point out an important difference in the levels of expression of *ASN* genes among mature leaves and young leaves of *L. japonicus* plants. A much higher level of expression was obtained for the *LjASN1* gene in mature leaves, compared to the young ones. This result would be consistent with a major role of the mature leaves as a source tissue for exporting asparagine to the young growing leaves of the plant, in accordance with the crucial involvement of asparagine as a N translocator in *L. japonicus* [2]. The high levels of *LjNSE1* asparaginase gene expression in young leaves indicated a high potential for asparagine utilization in this type of tissue of the plants. The fact that *LjNSE1* was the most highly expressed gene in all the plant tissues analysed, with the exception of nodules, suggested that the *LjNSE1* gene (which encodes a K^+^-dependent asparaginase) must be the most crucial gene for asparagine catabolism in leaves. Credali et al. [15] proved the crucial relevance of *LjNSE1* for plant growth and seed production of *L. japonicus*.

On the other hand, the fact that the expression of the *LjASN2* gene is restricted to the non-photosynthetic tissues examined, such as roots and nodules, suggests a crucial involvement of this gene for the particular metabolism of these organs. The very high levels of transcription of the *LjASN2* gene found in nodules seem particularly interesting since asparagine is one of the amino acids involved in the nitrogen export from the nodules. Prell and Poole [49] described that the amino acid cycling process is crucially relevant in symbiosis suggesting that, in addition to the exchange of carbon compounds and nitrogen fixed, plants and bacteroids also exchange amino acids. It is quite likely that the *LjASN2* gene may be crucial for the synthesis of asparagine in nodules, which is subsequently transported to the plant. In addition, the fact that only one particular asparaginase gene (*LjNSE2*) was expressed in nodules suggests an interesting role of this gene in nodule function.

The fact that *LjASN1* gene expression was shown to be light stimulated in *L. japonicus* (Fig. 1) is very interesting since class-I asparagine synthetases are usually up-regulated in the dark in response to a low carbon:nitrogen ratio. However, our results are in agreement with the previous data obtained by Waterhouse et al. [2] in the same plant, although in our case quantitative data of gene expression is provided. The light induction of *LjASN1* may be related with the fact that asparagine constitutes the major N-transport compound to undertake N-assimilation in *L. japonicus,* and nitrate reduction would be maximal in the light. In addition, in the present paper several lines of evidence establish clear links between *LjASN1* and photorespiration in *L. japonicus,* which could be taken as another possible explanation of the light inducibility of *LjASN1* gene*.* In fact, gene expression analysis (Figs. 8 and 9) indicated that the transcriptional regulation of *LjASN1*, together with those of *LjSGAT1* and *LjSGAT2*, were very similar to photorespiratory genes and quite different to other examined genes. Moreover, a further link between asparagine metabolism and photorespiration would come from the dramatic decrease in the expression of both *LjSGAT1* and *LjSGAT2* genes that occurs in the *Ljnse1–4* asparaginase deficient mutant from *L. japonicus* (Fig. 4). This mutant was shown to have much higher levels of asparagine [15]. Changes in SGAT expression in this asparaginase mutant could be related to the fact that asparagine plays an important role on the regulation of N flux in the N-organic pool [50] and that Modde et al. [51] have recently concluded that SGAT needs to be dynamically adjusted to ensure a variable flux through the photorespiratory pathway.

If there is any possible role of *LjASN2* in relation to photorespiration remains intriguing. The pattern of expression of *LjASN2* gene seems to be completely different to those of *LjASN1* and *LjSGAT* genes (Figs. 1, 8 and 9), thus not showing a clear link with photorespiration. Furthermore, the work carried out by Gaufichon et al. [9] with *ASN2* mutants argued that this gene does not contribute to photorespiratory N cycle. However, Fig. 5 shows that *LjASN2* is increased on the transfer from high-CO_2_ (suppressed photorespiration) to normal air (active photorespiration) conditions in the photorespiratory mutant *Ljgln2–2* lacking plastidic GS. Pérez-Delgado et al. [52] have shown that there is a strong increase in *LjASN2* gene expression, concomitant with the increase in photorespiratory ammonium produced as a result of the lack of plastidic GS. This increase in *LjASN2* gene expression takes place in parallel with an increase in asparagine levels in the same tissues that occurs simultaneously with an increase in glutamine due to the induction of cytosolic GS1 in the mutant [52]. It was concluded that this increase in *LjASN2* and asparagine forms part of the different responses of the plant to detoxify the high levels of photorespiratory ammonium produced by the absence of plastidic GS. A good correlation between *ASN2* gene expression and asparagine content was also found in *Arabidopsis*. The asparagine contents in *ASN2*- overexpressing and underexpressing plants were increased and decreased, respectively, when they were grown under normal light and nutrient conditions, suggesting that *ASN2* functions as a regulator of asparagine biosynthesis and metabolism and that it mediates the effective use of nitrogen under ammonium sufficient conditions [53]. Other types of evidence have proposed that *ASN2* is induced by ammonium treatments and by stress treatments that cause ammonium accumulation, suggesting a physiological role of *ASN2* related, directly or indirectly, to the reassimilation of the nitrogen remobilized under stress conditions [7]. A possible relationship between the level of *ASN2* gene expression and the level of ammonium loss via the photorespiratory pathway has been also described [7]. Several authors have reported the possibility of ammonium dependent synthesis of asparagine in plants [4].

## Conclusions

The results obtained in this paper indicate the existence of a differential expression of asparagine metabolic genes in *L. japonicus* and point out the crucial relevance of particular genes in different organs. Moreover, the data presented establish clear links between asparagine and photorespiratory metabolic genes in this plant.

## Additional files

Additional file 1: Table S1. Sequences of primers used in qRT-PCR experiments. (DOC 35 kb)

Additional file 2: Figure. S1. Phylogenetic tree of asparagine synthetases. A dendrogram of asparagine synthetase sequences was generated by PILEUP as previously described [5] including *LjASN1*, *LjASN2* and *LjASN3* from *L. japonicus.* Class-I and class-II phylogenetic clades are shown. (TIFF 626 kb)

Additional file 3: Figure. S2. Structures of the *LjASN*, *LjNSE* and *LjSGAT* genes from *L. japonicus.* Exons are represented as boxes. (TIFF 134 kb)

Additional file 4: Table S2. List and description of the *L. japonicus* transcriptomic data analyzed in the present work. All transcriptomic data are available online in the Arrayexpress database. (XLSX 14 kb)

## Abbreviations

ASN: asparagine synthetase ATP adenosine triphosphate GS1 cytosolic glutamine synthetase GS2 plastidic glutamine synthetase *LjGPI* *L. japonicus* glycosylphosphatidyl inositol-anchored protein *LjHAR1* *L. japonicus* hypernodulation aberrant root formation *LjPp2A* *L. japonicus* protein phosphatase 2A *LjUbc10* *L. japonicus* ubiquitin carrier protein 10 *LjUbq* *L. japonicus* polyubiquitin 4 NADH-GOGAT NADH-dependent glutamate synthase NiR nitrite reductase NR nitrate reductase NSE asparaginase qRT-PCR quantitative real-time polymerase chain reaction SGAT serine-glyoxylate aminotransferase TILLING Targeted Induced Local Lesions IN Genomes WT wild type
