# Supplementary material for: Genes for asparagine metabolism in Lotus japonicus: differential expression and interconnection with photorespiration
Source: BMC Genomics. 2017 Oct 12;18:781. doi: 10.1186/s12864-017-4200-x (PMC5639745; doi:10.1186/s12864-017-4200-x)
Supplement: Supplementary file 1 — Sequences of primers used in qRT-PCR experiments. (DOC 35 kb) [file 12864_2017_4200_MOESM1_ESM.doc]

Supplementary Table S1. Sequences of primers used in qRT-PCR experiments.

|  | Forward | Reverse |
| --- | --- | --- |
| *LjNSE1* | ATGCCAATCAGTGTGTACGCC | GTCCACTACCACGCACCCC |
| *LjNSE2* | TGTGGAGCTGTCTCTGGCCT | TGACCAATCGAGCCAAGGAA |
| *LjNSE3* | ACACGGCTGGATGAAGGGA | AGGCCACTTCACCCTTATTCG |
| *LjASN1* | TGGAGGACCAACTGTTGCATG | AGACCAAGCAGCATCCCACTC |
| *LjASN2* | TCAGTGAGCAAAGGTGTTGAACC | CAAGAGGAGAAACTTCCATCTTGG |
| *LjASN3* | CGAACTGGCAGTGATTGTGAAGTG | ATGCCAATAGCATCACGAGCAG |
| *LjSGAT1* | TCTCCAAGATGTGGGTTATCCTGT | GTATGCACTGGCAGCAGCAA |
| *LjSGAT2* | ACCCGGACGACACATTCTGTT | CCCGATCACTTTGTCAGGGAT |
| *LjHAR1* | TCATTTTCCATATTGGTCCTTCG | GAATTGTTCCTGTAATGGGTTTGG |
| *LjGAPDH5’* | AAGGATCGGGCGTTTGG | AGCAACAAGTTCAACATCGTCTCT |
| *LjGAPDH3’* | CGGTTACACTGAAGATGATGTGG | GATACTTGACCTGTTGTCGCCA |
| *LjGPI-ap* | AGGTTGTTCCGTGAATTTCG | GGTCCTTTGCATTTGCTTGT |
| *LjPP2A* | TGAGCTATGTGAAGCTGTTGGT | CAGCCTCATTATCACGCAGTAG |
| *LjUbc10* | GCTCTTATCAAGGGACCATCAG | ACTGCTCTGGAACAGAAAAAGC |
| *LjUbq4* | TTCACCTTGTGCTCCGTCTTC | AACAACAGCACACACAGACAATCC |
