# Supplementary figures and images for: Genes for asparagine metabolism in Lotus japonicus: differential expression and interconnection with photorespiration
Source: BMC Genomics. 2017 Oct 12;18:781. doi: 10.1186/s12864-017-4200-x (PMC5639745; doi:10.1186/s12864-017-4200-x)

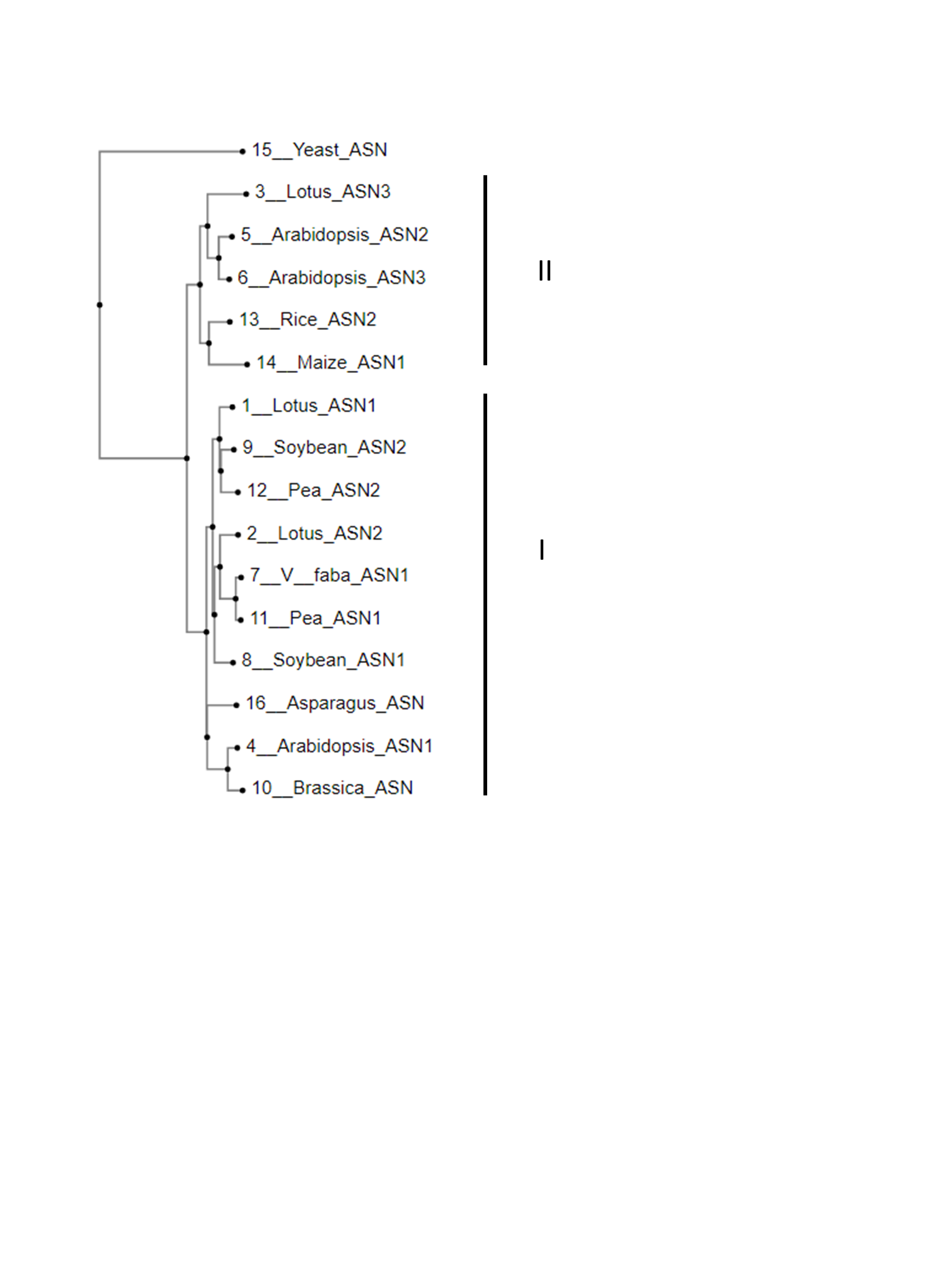

Supplement: Supplementary file 2 — Phylogenetic tree of asparagine synthetases. A dendrogram of asparagine synthetase sequences was generated by PILEUP as previously described [5] including LjASN1, LjASN2 and LjASN3 from L. japonicus. Class-I and class-II phylogenetic clades are shown. (TIFF 626 kb) [file 12864_2017_4200_MOESM2_ESM.tif]

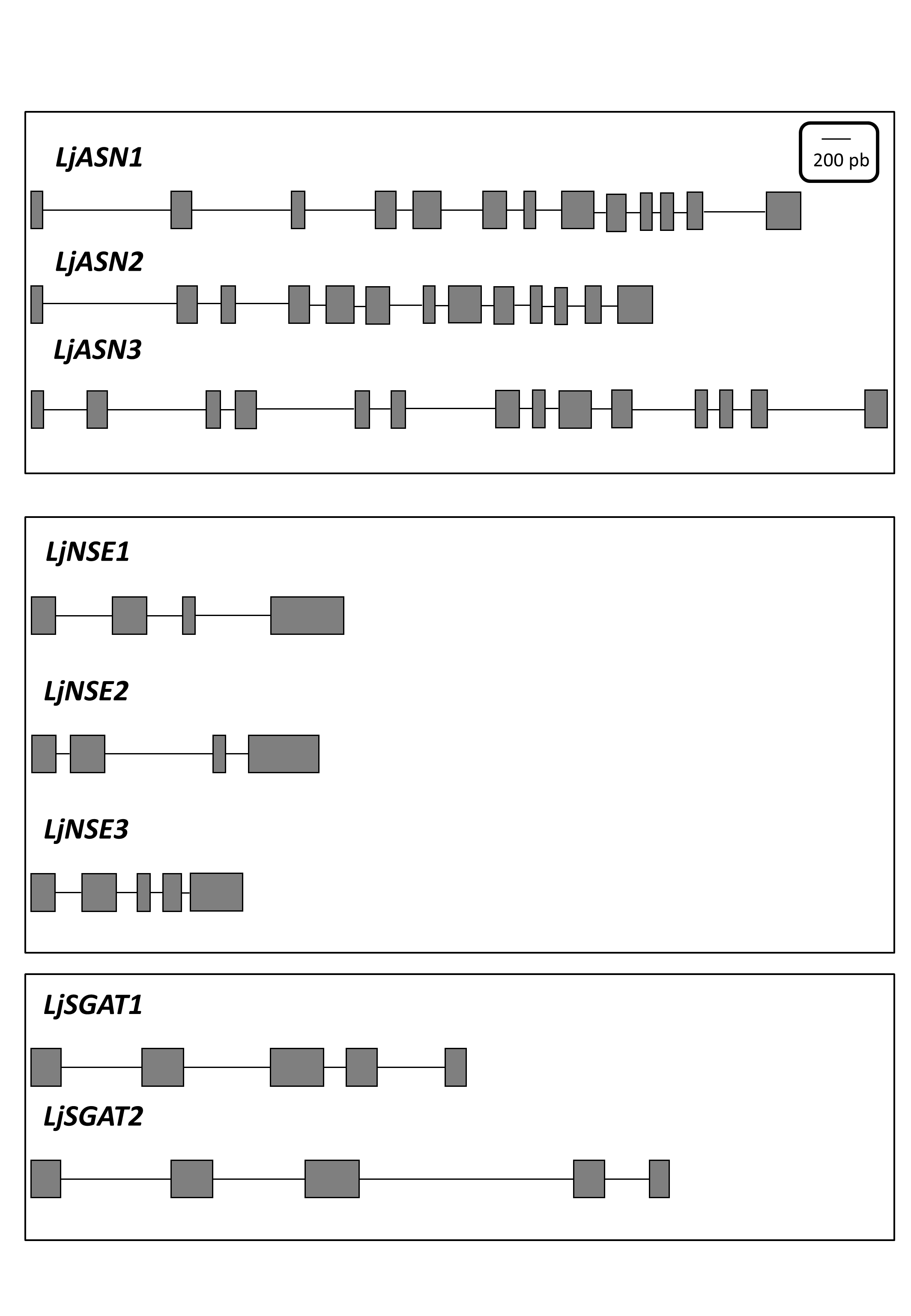

Supplement: Supplementary file 3 — Structures of the LjASN, LjNSE and LjSGAT genes from L. japonicus. Exons are represented as boxes. (TIFF 134 kb) [file 12864_2017_4200_MOESM3_ESM.tif]
